# Longitudinal Development of Reasons for Living and Dying With Suicide Attempters: A 2-Year Follow-Up Study

**DOI:** 10.3389/fpsyt.2022.865831

**Published:** 2022-05-12

**Authors:** Anja C. Gysin-Maillart, Rahel Jansen, Sebastian Walther, David A. Jobes, Jeannette Brodbeck, Simon Marmet

**Affiliations:** ^1^Translational Research Centre, University Hospital of Psychiatry and Psychotherapy, University of Bern, Bern, Switzerland; ^2^Unit for Clinical Suicide Research, Department of Clinical Sciences, Psychiatry, Faculty of Medicine, Lund University, Lund, Sweden; ^3^Department of Medical Psychology and Medical Sociology, University of Leipzig, Leipzig, Germany; ^4^Department of Psychology, The Catholic University of America, Washington, DC, United States; ^5^Institute for Counseling, Coaching and Social Management, School of Social Work, University of Applied Sciences and Arts Northwestern Switzerland, Olten, Switzerland; ^6^Department for Clinical Psychology and Psychotherapy, University of Bern, Bern, Switzerland

**Keywords:** reasons for living (RFL), reasons for dying (RFD), suicide attempts, suicidal ideation, Attempted Suicide Short Intervention Program (ASSIP)

## Abstract

**Background:**

Clinical interventions for patients after a suicide attempt might include a focus on Reasons for Living (RFL) and/or Reasons for Dying (RFD). The present study examined the longitudinal development of RFL and RFD in patients with and without a suicide-specific intervention - the Attempted Suicide Short Intervention Program (ASSIP).

**Methods:**

In this secondary analysis of a 2-year follow-up randomized controlled study, participants completed the Suicide Status Form II to assess RFL and RFD, at baseline, as well as at 6-, 12-, 18-, and 24-months follow-up. Growth models and latent class analysis were used to investigate longitudinal developments in RFL and RFD. Regression models were used to test the association between RFL, RFD and suicidal reattempts and ideation.

**Results:**

Cross-sectionally and longitudinally, RFD, but not RFL, were associated with suicide reattempts and suicidal ideation. The number of RFD decreased significantly across the 24 month period (from 1.90 at t1 to 1.04 at t5 in the control group and from 2.32 at t1 to 0.51 at t5 in the intervention group), and this decrease was stronger (b = −0.02; p = 0.004) in the ASSIP group than in the control group. There was no overall change in RFL. Three latent trajectories of RFD were identified: a decreasing (*n* = 77), a steady high (*n* = 17) and a trajectory with first increasing and then decreasing RFD (*n* = 26). The proportion of patients in the ASSIP intervention was highest in the decreasing trajectory and lowest in the steady high trajectory. Patients in the steady high trajectory were characterized by worse mental health and fewer social obligations (partner, children) at baseline.

**Conclusion:**

The results confirm the importance of RFD within the suicidal process and show that the number of RFD can be further reduced over the period of 24 months with short interventions such as ASSIP. The relevance of number of RFL in the suicidal process, as protective factor, was not confirmed. In the subgroup of patients whose RFD did not decrease over a long period of time, there is a particularly high risk of suicidal ideation/behavior. Clinical interventions should focus more closely on RFD, their etiology and maintenance.

## Introduction

With more than 700,000 deaths per year, suicide is a serious global health problem. The Swiss incidence for suicide in 2018 was 12 per 100,000 inhabitants, which is within the cross-national mean range (1, 2). Despite extensive research on risk factors, prediction of suicide remains difficult (3). A past suicide attempt is one of the greatest risk factors for subsequent suicide attempts (4, 5). This risk is especially high during the period immediately after the index attempt (6) - with 80% of subsequent suicide deaths occurring within 1 year (7). Another risk factor is suicidal ideation, which significantly increases the risk for suicide attempts and death (8). Approximately 30% of people with suicidal ideation subsequently attempt suicide and around two thirds of these transitions occur within a year after the initial emergence of suicidal thoughts (9). It is therefore crucial to understand the motivational mechanisms leading from suicidal ideation to suicidal behavior (10).

Within their internal struggle hypothesis of suicide, Kovacs and Beck (11) suggested that suicidal individuals experience inner ambivalence between life and death. Suicidal behavior is one potential result of this struggle between the concurrent wish to live (WTL) and wish to die (WTD). In fact, predominance of WTD over WTL has been identified as a risk factor for suicide (12), whereas higher levels of ambivalence between the two may lower suicidal intent (11).

Since wishes may lie beyond a patient's awareness, Jobes and Mann (13) argued that looking at concrete reasons that draw a patient to life or death may lead to a more valid risk assessment. The Reasons for Living Inventory (RFL) by Linehan et al. (14) had been developed more than 15 years before and assesses life-oriented beliefs that may keep people from suicide and which reliably differentiate between suicidal and non-suicidal samples. Subsequent findings suggest that RFL is a protective factor for suicidal ideation/behavior and its correlates (15–17). On the other hand, prospective studies have shown that persons with few RFL have an increased risk of developing suicidal ideation (18) and of attempting suicide (19).

By incorporating both sides of the suicidal equation, Jobes and Mann (13) developed the Reasons for Living (RFL) and Reasons for Dying (RFD). Assessment, a qualitative measure to categorize individual reasons that draw patients to life or to death. Harris et al. (20) used this assessment in an anonymous online survey, that classified participants as highly suicidal vs. non-suicidal on the basis of the Suicide Behavior Questionnaire-Revised (SBQ-R) (21) and WTL/WTD Scores. 94.1% of the highly suicidal group but only 22.4% of the non-suicidal group reported that they were engaging in a life vs. death debate. Not only did the highly suicidal group exhibit significantly fewer RFL and significantly more RFD than the non-suicidal group, but they also reported significantly more RFD than RFL. This finding is supported by a very recent study in a sample of military psychiatric inpatients (22) that found an association between greater numbers of reported RFD relative to RFL and greater hopelessness, as well as a history of multiple suicide attempts.

Other findings suggest that RFD may be more important than RFL as a measure of suicidal thoughts and behavior. In a community sample across the suicidal spectrum, RFD were indeed strongly associated with suicidal symptoms as assessed by the Suicidal Affect-Behavior-Cognition Scale (SABCS) and RFD variables explained 26% of variance in the SABCS score (23). Brüdern et al. (24) performed a secondary analysis of a 2-year follow-up study based on the patients who were allocated to the control group of the present study, and showed that participants after a suicide attempt reported significantly more RFL than RFD. At the same time, the number of RFL was not associated with depression at baseline, nor suicide ideation and repeated attempts over time. On the other hand, higher numbers of RFD were associated with both higher levels of depression at baseline and suicide ideation over the course of the 2-year follow-up. Finally, higher RFD scores were listed by participants with a history of suicide attempts than by participants without prior attempts. Although RFL seem to be a protective factor in individuals with suicide ideations, their influence on suicidal thoughts and behavior in attempters might be limited (24).

Given these findings, it is crucial to further explore RFD in interventions targeting the high-risk group of individuals with a history of suicide attempts. With the exception of (24), the influence of RFL and RFD on suicidal ideation/behavior has not been investigated longitudinally. Furthermore, no publication of longitudinal data exists to better examine the development of RFL/RFD in a suicide-specific treatment and a control group.

The present study aims to investigate and compare the longitudinal development of the number of RFL and RFD in patients who attempted suicide and received either a single session suicide risk assessment or a suicide specific intervention, both in addition to treatment as usual. From a variable-centered perspective, we firstly investigated whether there were overall changes in RFD and RFL over time and whether this differed between the control and intervention groups. We hypothesized that participants in the ASSIP group would report significantly more RFL and significantly fewer RFD over 24 months than participants in the control group. These variable-centered approaches are optimal for describing the development in the overall sample and test associations between variables. Secondly, we used a person-centered approach to identify and describe distinct trajectories of RFD and RFL over time. Thirdly we explored how RFL and RFD were associated with current and future suicidal ideation and behavior.

## Methods

### Procedure

The present study was part of a randomized clinical trail to evaluate the effectiveness of ASSIP (25), incorporating an intervention and a clinical control group. In this secondary analysis, data were analyzed from the randomized controlled trial of ASSIP (25). Study procedure was approved by the Ethics Committee of Bern in accordance with the Declaration of Helsinki (26) (register number 144/08, trial registration: ClinicalTrials.gov NCT02505373). The participants in the intervention group (n = 60) took part in the Attempted Suicide Short Intervention Program (ASSIP). Participants in the control group (*n* = 60) underwent a single clinical interview based on the SSF-II (27), in order to assess suicide risk. Both groups continued treatment as usual (TAU). Participants filled out a set of questionnaires after the initial session or the clinical interview and at 6, 12, 18, and 24 months.

### Participants

Participation was restricted to persons admitted to the emergency unit of the General University Hospital in Bern, Switzerland after a suicide attempt, and who were asked to participate in this randomized controlled study (25). If informed consent was given, participants were randomly allocated to either the intervention (ASSIP) or to the control group (see below). Suicidal behavior was defined as self-inflicted, potentially injurious behavior with a non-fatal outcome, but with either explicit or implicit intent to die (28). Serious cognitive impairment, insufficient mastery of the German language, psychotic disorder, and residency outside the hospital catchment area were considered as exclusion criteria. Diagnostic information was gained through hospital diagnosis based on the 10th revision of the International Classification of Diseases (29).

### Attempted Suicide Short Intervention Program (ASSIP)

The brief therapy ASSIP is a specific intervention for patients after attempted suicide, and is based on a patient-centered model of suicidal behavior (25, 30). Suicide may appear as an option to escape from a subjectively unbearable life situation and may repeatedly (and increasingly) emerge throughout life as a possible coping strategy when major life or identity goals are seriously threatened.

In the first ASSIP session, the so-called narrative interview, the therapist aims to build a shared understanding of the patient's suicidal story and establish an early therapeutic relationship. At the end of the first (or second) session, participants are asked to read the psychoeducational handout *Suicide is Not a Rational Act* (31, 32). In the second session, patient and therapist jointly re-watch sequences of the video-recorded narrative interview. While in a safe environment, aspects of the suicidal process, such as automatic thoughts, emotions, behaviors, and physiological changes present during the suicidal crisis are analyzed and individual vulnerabilities as well as threatened life-goals/needs are explored. Furthermore, individual warning signs are revealed and personal safety strategies are developed. In the third session, patient and therapist collaboratively revise the written summery of the case conceptualization. The patient receives a leporello the size of a credit card - in order to carry the safety plan with him/her. Once the face-to-face appointments have been completed, patients receive regular semi-standardized letters over the course of 2 years. The letters should remind them of their safety strategies and long-term (therapy) goals. Furthermore, they aim to maintain an ongoing alliance to patients and provide an easy access to the health care system.

### Measures

#### Assessment of Reasons for Living (RFL) and Reasons for Dying (RFD)

RFL and RFD were assessed as part of the Suicide Status Form (SSF-II) (27). The questionnaire was completed collaboratively by patient and therapist. Patients were asked to write down up to five RFL and RFD each (in the space provided), under the guidance of the therapist. Follow-up measurements were sent to the patient's home, with the instruction to fill in the RFL and RFD on the SSF-II individually. For the current analyses, the numbers of RFL and RFD responses were reported.

#### Sociodemographic Questionnaire (DEMO)

Personal and sociodemographic characteristics were collected with a 33-item questionnaire developed by the study team (25). The DEMO covers various health-related topics, including previous suicide attempts, suicidal behavior, and suicide ideation. Information about suicidal behavior and completed suicide was gathered by searching hospital records and contacting the patient's general practitioners and therapists.

#### Beck Scale for Suicide Ideation (BSS)

The Beck Scale for Suicide Ideation (33) is a 21-item self-report instrument assessing various aspects of suicide ideation, including attitudes, behaviors, and plans related to suicidal behavior. Each item consists of three statements representing a 3-point Likert scale (0 = not existing to 3 = severe), describing different intensities of suicide ideation. If item four (no indication of active suicidal intention) and five (indication of avoidance of death if resented with a life-threatening situation) were endorsed as 0 = not existing, the participant skipped the next 14 items, which address specific information about the respondent's plans and attitudes. Items 20 and 21 are qualitative items and were not included in the total score. A sum score over all items except for the last two items is built, with higher total scores representing higher suicide risk, although there is no cutoff score to distinguish between different risk categories (total score ranges from zero to 38). Kliem et al. (34) reported very good internal consistency for the German version of the BSS - with a Cronbach's alpha of 0.94.

#### Beck Depression Inventory (BDI)

The BDI assesses the severity of the patient's current level of depression. The 21 self-report items cover affective, cognitive, motivational, behavioral, and somatic components. All items are scored on a 4-point Likert scale (0 = not existent to 4 = severe). An overall sum score of 18 or above indicates significant depressive symptoms. The German version of the BDI has demonstrated good validity, as assessed by Hautzinger et al. (35).

### Statistical Analyses

Data preparation and descriptive statistics were implemented in SPSS 27. For descriptive statistics, differences in continuous variables were tested using a *t*-test, and differences in categorical variables with a chi-squared -test. To account for missing values, 100 multiple imputation data sets were computed using analyses based on Markov Chain Monte Carlo simulations in a Bayesian framework in Mplus 8 (36, 37).

Trends in the number of RFL and RFD were investigated using two different approaches: a variable-centered and a person-centered framework. Variable-centered approaches are ideal to investigate overall associations between variables, while person-centered approaches focus on identifying and describing relevant subgroups of individuals. For the variable-centered framework, longitudinal trends in the numbers of RFL and RFD were estimated in Mplus 8 using a linear growth model. This model estimates two parameters, the intercept, and the slope, with the intercept representing the initial level of RFD and the slope representing change in RFD or RFL over time (measured in months after baseline). Models were estimated separately for the control and the intervention group. Additionally, a model using the total sample was tested for differences in the slope parameter between the intervention and the control groups. This model was adjusted for the intercept at baseline, in order to account for differences in the number of RFD and RFL at baseline. Differences between the control and intervention groups in the number of RFL and RFD at each time point and differences between time points within groups were tested using the Wald test for parameter differences in Mplus 8.

For the person-centered approach (38, 39), an exploratory longitudinal latent class analysis was conducted in Mplus 8. Given that there were no changes over time in RFL, the model was estimated based on the number of RFD only. The aim of the latent class analysis was to identify distinct subgroups of participants with similar trajectories of RFD over time, and to investigate how these subgroups differ from other suicide and mental health related variables. Models with 2 to 5 latent classes were estimated and the optimal solution was chosen, as based on statistical indicators (BIC, AIC, and LRT tests) and the clinical usefulness of the solution. Models were estimated based on the basis of 100 multiple imputation data set. Mean or proportions for auxiliary variables were calculated using the DCON or DCAT commands, respectively (40).

To test associations between the number of RFD and RFL with suicidal ideation, linear regression with the BSS score at t5 as the outcome was performed for the total sample. Predictors were the number of RFD and RFL at t1 to t5.

To test associations between the numbers of RFD and RFL with suicide reattempts, ordinal logistic regression models were employed with the number of suicide reattempts (capped at 3) over the 24-month period as dependent variables. Models for the numbers of RFL and RFD at t1 to t5 were estimated. For each time point of RFL and RFD, the outcome was the sum of attempts in the current and in the future periods, e.g. t2 to t5 for RFD at t2 and t4 to t5 for RFD at t4.

## Results

### Participant Characteristics

Between January 2009 and December 2012, 54 (45%) male and 66 (55%) female participants with a mean age of 37.8 years were included in the study. Out of a total of 120 patients, 30 (25%) participants were diagnosed with substance use disorder, 76 (63%) with affective disorder, 53 (44%) with a neurotic and acute stress reaction and 20 (17%) showed a personality disorder. Half of the participants (50%) were admitted after a first suicide attempt and 26 percent had a history of multiple attempts. At baseline, comparison of intervention and control group showed no significant differences in terms of demographic and clinical variables (Table 1).

**Table 1:** Sociodemographic and clinical characteristics of study participants at baseline.

	**ASSIP**	**Control**	**All Participants**	***p*-value**
	***N* = 60**	***N* = 60**	***N* = 120**	
Gender (Male/Female) – *n* (%)	24 (40)/36 (60)	30 (50)/30 (50)	54 (45)/66 (55)	0.36^a^
Age (years) – *M* (*SD*)	36.5 (14.3)	39.2 (14.6)	37.8 (14.4)	0.32^b^
Diagnosis^c^ (ICD−10) – *n (*%)				
F1 (substance use disorder)	10 (17)	20 (33)	30 (25)	0.06^a^
F3 (affective disorder)	40 (67)	36 (60)	76 (63)	0.57^a^
F4 (neurotic and acute stress reaction)	25 (42)	28 (47)	53 (44)	0.71^a^
F6 (personality disorder)	8 (13)	12 (20)	20 (17)	0.46^a^
Others	6 (10)	1 (2)	7 (6)	0.12^a^
Married – n (%)	19 (32)	15 (25)	34 (28)	0.54^a^
Children – n (%)	25 (42)	19 (32)	44 (37)	0.34^a^
Living Alone – n (%)	20 (33)	23 (38)	43 (36)	0.70^a^
Employed – n (%)	34 (57)	36 (60)	70 (58)	0.85^a^
Prior Suicide Attempts – n (%)				
0	34 (57)	26 (43)	60 (50)	0.71^a^
1	16 (27)	13 (22)	29 (24)	
2 or more (multiple)	10 (17)	21 (35)	31 (26)	
BDI t1 – M (SD)	18.05 (11.45)	18.32 (12.25)	18.19 (11.81)	0.90^b^
BSS t1 – M (SD)	7.44 (8.43)	9.05 (9.15)	8.25 (8.80)	0.32^b^

a *χ^2^*.

b *t test*.

c *Totals exceed 100% because of multiple diagnoses. ICD: International Classification of Diseases*.

### Differences Between Groups in Longitudinal Development of Numbers of RFL and RFD

**Figure 1:**
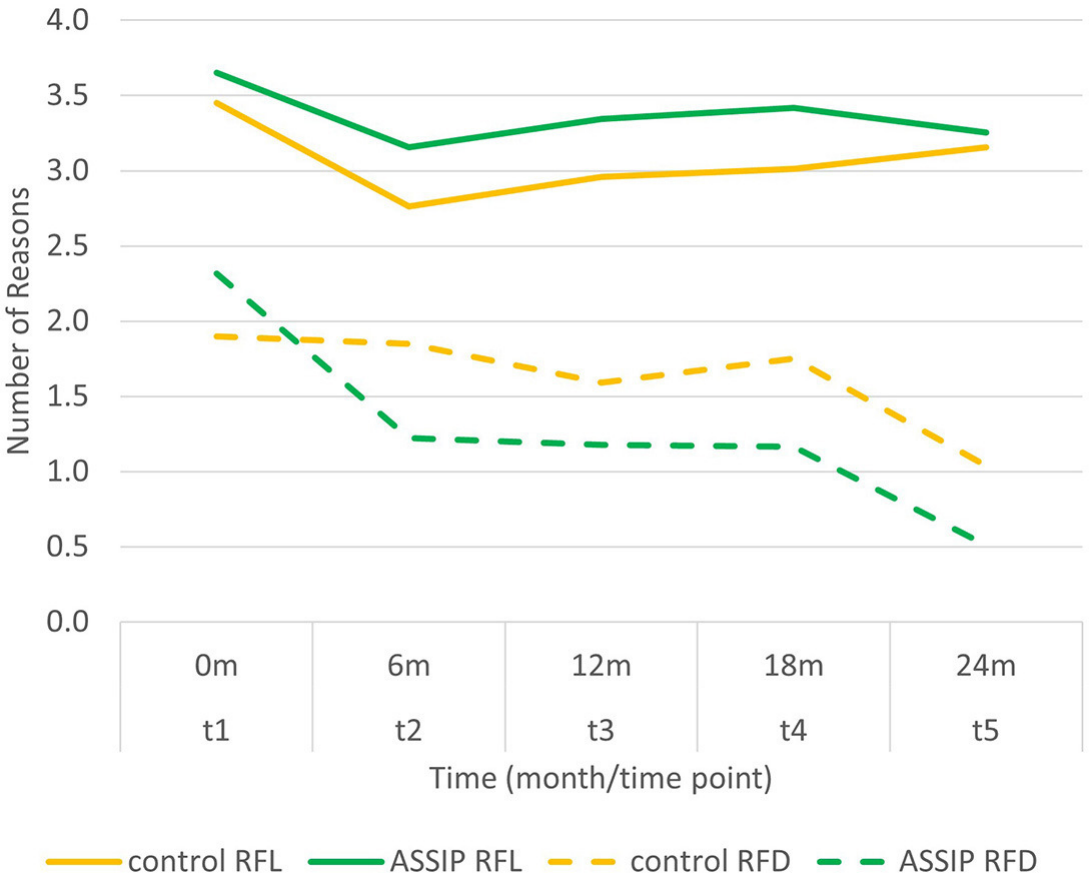
Mean number of reasons for living (RFL) and reasons for dying (RFD) over the course of the two-year follow-up.

Overall, there was no significant change in number of RFL over the entire 24 month period (mean at t1 = 3.55; at t5 = 3.21) in either the intervention or the control group. There was a slight decrease in number of RFL from t1 to t2, which reached significance for the control group (difference = −0.687; *p* = 0.01), but not in the intervention group (difference = −0.492; *p* = 0.052). There were no significant differences in number of RFL between any other time points. The intervention group had slightly higher numbers of RFL than the control group at all time points, but this did not reach significance at any time point (Figure 1, Table 2).

**Table 2:** Development of number of RFD/RFL over time.

	**% at least one reason/**					**Change over time**	
	**Mean (SD) number of reasons**					**(latent growth)**	
	**t1**	**t2**	**t3**	**t4**	**t5**	**Slope**	***p*-value**
**Reasons for Living (RFL)**
% at least 1 RFL							
Control	96.7%	87.7%	88.9%	89.6%	92.0%		
ASSIP	100.0%	89.4%	95.0%	92.3%	91.1%		
mean total RFL
Control	3.45 (1.33)	2.76 (1.74)	2.96 (1.65)	3.01 (1.57)	3.15 (1.57)	0.00	0.699
ASSIP	3.65 (1.17)	3.16 (1.66)	3.34 (1.49)	3.42 (1.59)	3.26 (1.47)	−0.01	0.227
difference ASSIP–control	0.20	0.40	0.38	0.41	0.11	0.01	0.665
**Reasons for Dying (RFD)**
% at least 1 RFD							
Control	73.3%	67.4%	67.0%	66.1%	48.7%		
ASSIP	78.3%	49.3%	49.8%	46.6%	28.9%		
mean total number
Control	1.90 (1.54)	1.85 (1.79)	1.59 (1.51)	1.75 (1.67)	1.04 (1.31)	**−0.03**	<0.001
ASSIP	2.32 (1.67)	1.22 (1.55)	1.18 (1.55)	1.16 (1.50)	0.51 (0.93)	**−0.05**	<0.001
difference ASSIP–control	0.42	**−0.63**	−0.41	−0.59	**−0.53**	**−0.02**	0.004

*Slope is change in number of reasons per month. Bold coefficients are significant at p < 0.05*.

In comparison to the control group, the number of RFD was higher (not significant) in the intervention group at t1, and lower at t2 to t5 (significant difference for t2 and t5, but not t3, and t4). There was an overall decrease in number of RFD in the control (from 1.90 at t1 to 1.04 at t5; slope = −0.03 reasons per month; *p* < 0.001) and in the intervention group (from 2.32 at t1 to 0.51 at t5; slope = −0.05 reasons per month; *p* < 0.001). There was a significant interaction between slope and treatment condition (control vs. intervention), i.e., the decrease in number of RFD was significantly stronger (difference = −0.02 reasons per month; *p* = 0.004) in the intervention group than in the control group. As regards the comparisons between time points, there were significant decreases in the number of RFD between t1 and t2 in the intervention group (difference = −1.094; *p* < 0.001) and between t4 and t5 in both the control (difference = −0.711; *p* = 0.001) and the intervention group (difference = −0.653; *p* = 0.002). The proportion of patients mentioning at least one RFD decreased from 73.3% at t1 to 48.9% at t5 in the control group and from 78.3% at t1 to 28.9% at t5 in the intervention group.

### Identification of Subgroups in the Longitudinal Development of RFD

**Figure 2:**
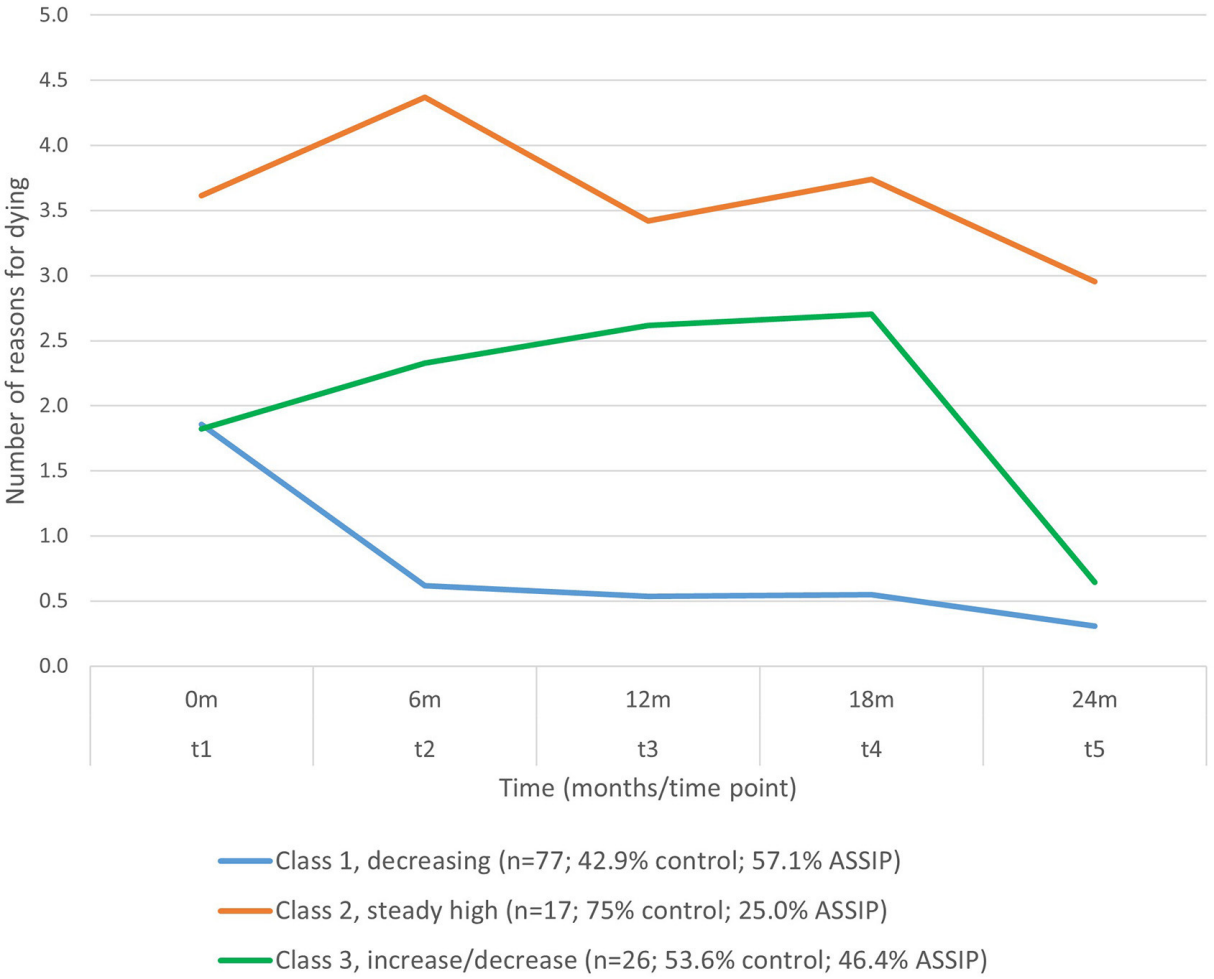
Latent trajectories of reasons for dying from t1 to t5.

An exploratory latent class model was estimated for trajectories of the number of RFD from t1 to t5. Solutions for 2 to 5 classes were calculated. On the basis of statistical indices (see Supplementary Table S1) and clinical meaningfulness, a three-class solution was retained (Figure 2). The largest class (*n* = 77) showed an overall decrease in the number of RFD and was labeled “decreasing” trajectory. At t1, number of RFD (1.86) was close to the sample mean for RFD (2.11). There was a marked decrease in number of RFD toward t2 (0.62), and a further decrease toward t5, where number of RFD at t5 was very low (mean = 0.31). In this largest class, 57.1% of participants were in the intervention group, and 42.9% in the control group (Table 3). The second class (*n* = 17) remained high in RFD from t1 to t5 and was labeled “steady high”. In this class, the proportion of individuals participating in the intervention was the lowest (25.0%). This second class also had the highest number of repeated suicide attempts across the period of 24 months and the highest levels of suicidal ideation at all time points (Table 3). A third class was identified that showed first a slight increase and then a decrease in number of RFD toward t5 (*n* = 26), and this group was labeled “increase/decrease”. In all three classes, there was a relatively marked decrease in the number of RFD from t4 to t5.

**Table 3:** Properties and covariates for the latent class solution.

Class size and proportion of ASSIP					
	*n* total	*n* control	*n* ASSIP	Control%	ASSIP%	
Class 1	77	33	44	42.9%	57.1%	
Class 2	17	13	4	75.0%	25.0%	
Class 3	26	14	12	53.6%	46.4%	
Reasons for dying					
	t1	t2	t3	t4	t5	
Class 1	1.86	0.62	0.54	0.55	0.31	
Class 2	3.61	4.37	3.42	3.74	2.95	
Class 3	1.82	2.33	2.62	2.70	0.64	
Reasons for living
	t1	t2	t3	t4	t5	
Class 1	3.60	2.71	3.09	2.95	3.04	
Class 2	3.30	3.57	3.51	3.82	3.98	
Class 3	3.57	3.25	3.08	3.67	3.19	
Suicidal ideation (BSS score)
	t1	t2	t3	t4	t5	
Class 1	4.66	1.40	1.18	1.43	1.10	
Class 2	16.97	19.53	12.40	12.58	9.01	
Class 3	11.64	9.67	10.22	8.69	4.59	
Suicide reattempts (number)
	Previous attempts	t2	t3	t4	t5	total 24 months
Class 1	0.92	0.03	0.03	0.04	0.12	0.22
Class 2	2.62	0.71	0.09	0.49	0.39	1.67
Class 3	1.17	0.30	0.15	0.07	0.10	0.62

Table 3 and Supplementary Table S2 show results for predictors measured at baseline: class 2 (steady high) showed the highest BDI scores at baseline (28.66 vs. 14.86 in class 1), the highest proportion of F6 diagnoses (42.9 vs. 8.1% in class 1), the highest numbers of prior suicide attempts (2.62 vs. 0.92 in class 1), and were the least likely to be married, to be in a relationship or to have children. In general, class 1 was the lowest for these factors, and class 3 was intermediate.

### Associations of RFL/RFD With Suicidal Ideation and Reattempts

The number of RFD were associated cross-sectionally (beta = 0.490 at t5) and longitudinally (beta between 0.193 and 0.490) with BSS score (Table 4). In general, the numbers of RFL showed a slightly negative association with suicidal ideation, but this only reached significance for the association between RFL at t3 and suicidal ideation at t5.

**Table 4:** Associations between RFD/RFL and suicidal ideation (BSS Score) and number of suicide reattempts.

	**Suicidal ideation**			**Suicide reattempts**		
	**(BSS T5)**			**(0–24 months)**		
	**Beta**	***p***	**R-square**	**OR**	***p***	**R-square**
Reasons for living						
RFL t1	−0.128	0.185	0.017	0.92	0.645	0.006
RFL t2	−0.092	0.347	0.010	0.91	0.514	0.011
RFL t3	**−0.195**	0.030	0.040	0.79	0.149	0.045
RFL t4	−0.045	0.661	0.004	0.94	0.743	0.011
RFL t5	−0.041	0.646	0.003	0.90	0.588	0.018
Reasons for dying						
RFD t1	**0.193**	0.031	0.038	1.30	0.062	0.054
RFD t2	**0.477**	<0.001	0.228	**1.34**	0.020	0.070
RFD t3	**0.463**	<0.001	0.215	1.25	0.121	0.037
RFD t4	**0.258**	0.008	0.068	1.29	0.112	0.053
RFD t5	**0.490**	<0.001	0.241	1.37	0.176	0.044

*Outcome for suicidal ideation is Beck Scale for Suicide Ideation (BSS). Bold coefficients are significant at p < 0.05. Model is linear for suicidal ideation and ordinal logistic regression for suicide reattempts. For suicide attempts, outcome is the sum of attempts in the current and future period, e.g. t2 to t5 for RFD at t2 and t4 to t5 for RFD at t4*.

Numbers of suicide reattempts were significantly lower in the intervention group, compared to the control group (OR = 0.16). The numbers of RFD at all time points were consistently associated with more suicide reattempts in the current and future periods (OR ranging from 1.25 to 1.37; Table 4). However, this was significant only for RFD at t2. The number of RFL was consistently associated with fewer suicide attempts, but this negative association was much weaker than the positive association of RFD with ORs ranging from 0.79 to 0.94 (not significant).

## Discussion

In this present study, we aimed to investigate the development of the number of RFL and RFD during the course of the study period and to examine the associations of RFL/RFD with suicidal ideation/behavior.

### Development of RFL and RFD Over Time

Based on a variable-centered perspective, we first examined whether there were overall changes in the number of RFD and RFL over time and whether these differed between the control and intervention groups. Contrary to our assumption, the number of RFL did not increase in the ASSIP group over the 24-month period and the ASSIP and control group did not significantly differ in their number of RFL exhibited at any time point. Instead, the number of RFL decreased in both experimental groups between baseline and after 6 months (significant for the control group only). There was no significant change in the number of RFL across any other time point within groups. There are several explanations for the drop in number of RFL in both groups between baseline and after 6 months: according to Rudd et al. (41) a suicidal act can cause the suicidal mode to subside, and this is sometimes referred as a “cathartic effect” (42). However, there is no clear consensus on a suicidal catharsis effect for patients who have engaged in suicidal behavior. For example, Pompili et al. (43) found converse findings in patients admitted to an emergency department, with persistently high scores on suicide-related scales (e.g., suicidal ideation, reasons for death). Walker and colleagues (42) argue that apparent cathartic effects following suicidal behavior may actually be linked to the accrual of relational support. At baseline, after the suicide attempt, patients may have been in a mental state in which they had already regained the capacity to perceive their RFL. Additionally, most patients experience the narrative interview as tension relieving (31). A similar release of tension may also occur after the structured risk assessment and may enhance the capacity to reproduce RFL. This assumption is strengthened by our results with SFF-II – a collaborative questionnaire and part of the Collaborative Assessment and Management of Suicidality (CAMS) approach, which has been proven to be effective in reducing suicidal ideation (44) with its collaborative approach. Finally, most patients may have been willing to work on their suicidal ideation and behavior.

Although the numbers of RFD dropped significantly in both treatment arms over the course of the two-year follow-up, the decline began markedly earlier in the ASSIP group. Accordingly, the number of RFD was significantly lower in the ASSIP group than in the control group after 6 months, which was also apparent after 24 months. It therefore seems that rather than building up novel RFL, ASSIP targets and reduces existing RFD. The decline in RFD may be associated with a potential mechanism of ASSIP: threatened life-goals and vulnerabilities are revealed through the narrative interview and later during the video playback. The self-confrontation allows a controlled re-immersion of the patient into the so-called suicidal mode (45), without getting lost in it. This allows a cognitive and emotional exposure. The suicidal mode (45) is a cognitive-emotional-behavioral and psychological response pattern characterized by a mental narrowing on ending an unbearable condition, which individuals experience during the suicide attempts. In Video-Playback, patients gain an understanding of their personal suicidal crisis, in a biographical context, including mechanisms that brought them to the point of attempting suicide. Targeting RFD while being embedded in a secure therapeutic relationship could help to address these and ultimately lead to a decrease in the number of RFD. The decrease in the number of RFD after 24 months in the control group is also consistent with prior evidence that suicidal risk is especially high in the 2 years following the suicide attempt (46, 47). Similar to the findings of Brüdern et al. (24), there are now more data to support the explanation that RFL and RFD may represent two fundamentally different components of suicidal ideation and behavior rather than being two poles on a continuum.

### Identification of Subgroups of the Development of RFD

By using a person-centered approach, we investigated whether there are subgroups with distinct trajectories of the number of RFD and RFL over time, and whether these subgroups differ with respect to group membership (control vs. intervention), suicidal ideation, and behavior. While variable-centered approaches are very useful for testing overall associations between measures, they omit the fact there may be different and meaningful subgroups within the sample. Our latent class analysis identified three such subgroups, respectively classes: most participants in the control and the intervention group were in *class 1* and reduced their RFD over time after a suicide attempt, along with reduced suicidal ideation. Only a relatively small *class 2* (*n* = 17) still showed high level of RFD and suicidal ideation at all time points including t5, and this class accounted for the overall mean of RFD, suicidal ideation, and suicide reattempts in the control group and in the ASSIP intervention. Suicidal behavior is one potential result of this struggle between the concurrent wish to live (WTL) and wish to die (WTD). In fact, predominance of WTD over WTL has been identified as a risk factor for suicide (12) whereas higher levels of ambivalence between the two may lower suicidal intent (11). This group is of particular clinical relevance, as in this group neither TAU nor the ASSIP intervention could reduce the number of RFD. However, of note, only 25% of this group participated in the ASSIP intervention (i.e., more individuals decreased their RFD in the ASSIP group and ended up in a different class), thus explaining to some degree the overall lower levels of RFD and suicide reattempts in the ASSIP group. One possible interpretation of this effect may be that ASSIP, as described above, directly addresses RFD, using it to develop long-term measures and (therapy) goals, which can be treated in a longer-term therapy. For those patients with persistently high RFD, addressing vulnerabilities (e.g., I am a failure) and threatened life-goals (e.g., I want to be successful) may be persistently threatened. Clinical interventions in this high-risk group should pay particular attention to understanding such RFD, so that tailored interventions can be derived.

The third class (*class 3*) showed a non-linear pattern (*n* = 26), with a slight increase in the number of RFD in the first half of the study period, and a strong decrease toward the end of the study period. This result indicates the importance of long-term treatment since patients show a decrease in the number of RFD only after 1 year. It is notable that there was a decrease in the number of RFD toward the end of the study period in all three classes. The ability to identify such non-linear patterns demonstrates a major strength of the latent class approach and shows that not all patients follow a pathway of either improvement, stability, or deterioration, but that there are also trajectories in-between.

The three trajectories differed in several variables measured at baseline. Class 2 (steady high) exhibited steady high levels of RFD and had the highest levels of depression - followed by class 3 (increase/decrease: where RFD increased and then decreased). Class 2 also had the greatest number of prior suicide attempts, with class 3 (increase/decrease) lying between class 2 (steady high) and class 1 (decrease: decrease in RFD). In comparison to class 1 (decrease), individuals in class 2 (steady high) and class 3 (increase/decrease), were less likely to be married, to be in a relationship or to live with children. Thus, it appears that these well-known risk factors for psychopathology (3) increase the risk of an unfavorable trajectory after a suicide attempt and indicate how important it is to provide more (and perhaps more specific) support to suicidal patients with more mental health problems and lower levels of social support.

### Association Between the Number of RFL/RFD and Suicidal Ideation and Reattempts

In addition, we explored how the numbers of RFL and RFD were associated with current and future suicidal ideation and behavior. Overall, the number of RFD was associated with (higher) suicidal ideation. The number of RFD was also associated with (more) suicide reattempts, but due to small number of suicide attempts, especially in the later time points, this reached significance only for the number of RFD at t2. This association of number of RFD with suicidal ideation and a possible association with more suicidal reattempts provides further support for the importance of RFD within the suicidal process. These findings are in line with previous work from Brüdern et al. (24), who found that the number of RFD was the strongest predictor for suicidal ideation at baseline. However, correlation decreased over time (after 1 year), which may indicate that RFD and suicidal ideation are not the same constructs. Previous work on RFL vs. RFD has considered motivational variables and psychosocial correlates of suicide (e.g., escape, revenge, the wish to die, or the wish to be killed) as “reasons for dying” (RFD) and emphasize the importance of internal, self-oriented work that may be separate from relationally oriented (interpsychic) work (13). Overall RFD seem to have a substantial role on suicidal ideation and further research should be conducted to better understand both constructs.

The numbers of RFL generally showed a negative association with suicidal reattempts and suicidal ideations, but this was only marginally significant for RFL at time point 3 and only for suicidal ideation. In the ASSIP group, numbers of RFD were lower than in the control group.

Although statistical power is limited due to the sample size and small numbers of suicide reattempts, it is plausible that the reduction in RFD by the ASSIP intervention contributes to its overall effect on reducing suicide reattempts.

### Limitations

Key limitations of the ASSIP RCT have already been discussed in the work by Gysin-Maillart et al. (25) and Brüdern et al. (24) who investigated the same patient cohort. First of all, self-reported data were primarily used to assess repeated suicidal behavior. Discrepancy between self-reports and hospital-based data may lead to over- or under estimation of repeated suicidal behavior (48). To counteract this known problem, hospital records and health professionals were consulted to complement self-reported data.

Due to psychological and ethical reasons, baseline assessment was conducted after the first ASSIP session or clinical interview, which may have had an impact on questionnaire responses. Furthermore, the clinical assessment in the control group was provided by ASSIP therapists and may have incorporated narrative aspects. The same therapists stood in minimal contact with control participants by sending them follow-up questionnaires with a personally signed letter. This kind of contamination may have resulted in a therapy-like relationship, that potentially could have reduced group differences in outcome measures.

We could only investigate the total number of RFL and RFD. However, other aspects such as strength and other qualitative characteristics of RFD and RFL may also be important in the suicidal process in addition to the number of reasons. Further research should investigate these characteristics in more depth.

Dropout rates and missing data are a common problem of long-term follow-up studies. Dropout rates were higher in the control group than in the ASSIP group and increased over the course of the two-year follow-up. To counteract this issue, analyses were conducted based on intention to treat (ITT). Missing data were addressed by using multiple imputations, which reduce bias due to missing outcome data, but may lead to a more conservative treatment effect.

Statistical power was limited due to the small sample size in this study, especially for suicide reattempts, which are relatively rare in the later time points. Future studies investigating similar research questions should therefore include greater sample sizes.

Finally, participants were instructed to name up to five RFL and RFD, which may have resulted in a ceiling effect.

## Conclusion

The findings of the present study indicate that the focus on protective factors (RFL) is not sufficient to reduce the risk of suicide. Clinicians may be tempted to over-emphasize RFL whereas the patient may benefit more by focusing on RFD. Understanding a suicidal crisis (and RFD) in a biographical context allows a change in perspective and helps to encourage work on underlying vulnerabilities. The results show that the number of RFD can be reduced further over the period of 24 months with short interventions such as ASSIP. In addition, future work needs to be performed on patients for whom RFD do not decrease over time, as this group is at high risk for future suicidal behavior. Clinical interventions should focus more closely on RFD.

## Data Availability Statement

Dataset can be made available upon request. Requests to access these datasets should be directed to anja.gysin@upd.unibe.ch.

## Ethics Statement

The studies involving human participants were reviewed and approved by Ethics Committee of Bern in accordance with the Declaration of Helsinki (35) (register number 144/08, trial registration: ClinicalTrials.gov NCT02505373). The patients/participants provided their written informed consent to participate in this study.

## Author Contributions

AGM conceived and designed the experiments, enrolled patients, and performed the experiments. SM, AGM, and RJ analyzed the data. AGM and RJ wrote the first draft of the manuscript. AGM, SM, RJ, SW, DJ, and JB contributed to the writing of the manuscript and agree with the manuscript's results and conclusions. All authors contributed to the article and approved the submitted version.

## Conflict of Interest

The authors declare that the research was conducted in the absence of any commercial or financial relationships that could be construed as a potential conflict of interest.

## Publisher's Note

All claims expressed in this article are solely those of the authors and do not necessarily represent those of their affiliated organizations, or those of the publisher, the editors and the reviewers. Any product that may be evaluated in this article, or claim that may be made by its manufacturer, is not guaranteed or endorsed by the publisher.
