# Supplementary material for: Longitudinal Development of Reasons for Living and Dying With Suicide Attempters: A 2-Year Follow-Up Study
Source: Front Psychiatry. 2022 May 12;13:865831. doi: 10.3389/fpsyt.2022.865831 (PMC9133363; doi:10.3389/fpsyt.2022.865831)
Supplement: Supplementary file 1 [file Data_Sheet_1.PDF]

Supplementary materials

Table S1. Information criteria and entropy for latent class solutions 2 to 5

Number of Classes	AIC	BIC	Entropy	n smallest class
2	2001.707	2046.307	0.948	27
3	1945.719	2007.044	0.92	17
4	1901.896	1979.946	0.934	15
5	1867.587	1962.361	0.954	7

Note: AIC: Akaike Information Criteria BIC: Bayesian Information Criteria

Table S2. Properties of the latent trajectory classes: sociodemographic factors, depression, and prior psychiatric diagnosis

**Sociodemographics**						
	Sex (%male)	age	Employment (%yes)	partner (%yes)	married (% yes)	child (%yes)
Class 1	45.1%	38.93	59.2%	55.9%	41.5%	47.6%
Class 2	33.9%	35.75	51.8%	26.0%	1.4%	23.4%
Class 3	52.6%	35.86	60.8%	23.7%	8.1%	11.1%
**Depression (BDI) score**						
	t1	t2	t3	t4	t5	
Class 1	14.86	7.83	7.55	6.31	4.00	
Class 2	28.66	26.39	20.46	25.69	19.46	
Class 3	21.31	20.57	17.00	22.69	12.45	
**Prior diagnosis (ICD-10 codes)**						
	F1	F3	F4	F6	others	
Class 1	25.9%	59.6%	48.8%	8.1%	6.6%	
Class 2	22.8%	70.1%	34.2%	42.9%	7.6%	
Class 3	25.4%	68.8%	37.9%	23.0%	1.7%	

(ICD-10) codes: F1*, substance abuse disorder; F3*, affective disorder; F4*, neurotic and acute stress reaction; F6*, personality disorder.
